# Motor Vehicle Crashes in Diabetic Patients with Tight Glycemic Control: A Population-based Case Control Analysis

**DOI:** 10.1371/journal.pmed.1000192

**Published:** 2009-12-08

**Authors:** Donald A. Redelmeier, Anne B. Kenshole, Joel G. Ray

**Affiliations:** 1 Department of Medicine, University of Toronto, Toronto, Ontario, Canada; 2 Clinical Epidemiology and Health Care Research Program, Sunnybrook Health Sciences Centre, Toronto, Ontario, Canada; 3 Institute for Clinical Evaluative Sciences in Ontario, Toronto, Ontario, Canada; 4 Patient Safety Service of the Sunnybrook Health Sciences Centre, Toronto, Ontario, Canada; 5 Division of General Internal Medicine, St. Michael's Hospital, Toronto, Ontario, Canada; 6 Division of Endocrinology, Women's College Hospital, Toronto, Ontario, Canada; Lund University Hospital, Sweden

## Abstract

**Background:**

Complications from diabetes mellitus can compromise a driver's ability to safely operate a motor vehicle, yet little is known about whether euglycemia predicts normal driving risks among adults with diabetes. We studied the association between glycosylated hemoglobin (HbA1c) and the risk of a motor vehicle crash using a population-based case control analysis.

**Methods and Findings:**

We identified consecutive drivers reported to vehicle licensing authorities between January 1, 2005 to January 1, 2007 who had a diagnosis of diabetes mellitus and a HbA1c documented. The risk of a crash was calculated taking into account potential confounders including blood glucose monitoring, complications, and treatments. A total of 57 patients were involved in a crash and 738 were not involved in a crash. The mean HbA1c was lower for those in a crash than controls (7.4% versus 7.9%, unpaired *t*-test, *p* = 0.019), equal to a 26% increase in the relative risk of a crash for each 1% reduction in HbA1c (odds ratio = 1.26, 95% confidence interval 1.03–1.54). The trend was evident across the range of HbA1c values and persisted after adjustment for measured confounders (odds ratio = 1.25, 95% confidence interval 1.02–1.55). The two other significant risk factors for a crash were a history of severe hypoglycemia requiring outside assistance (odds ratio = 4.07, 95% confidence interval 2.35–7.04) and later age at diabetes diagnosis (odds ratio per decade = 1.29, 95% confidence interval 1.07–1.57).

**Conclusions:**

In this selected population, tighter glycemic control, as measured by the HbA1c, is associated with an increased risk of a motor vehicle crash.

*Please see later in the article for the Editors' Summary*

## Abstract 2

Using a population-based case control analysis, Donald Redelmeier and colleagues found that tighter glycemic control, as measured by the HbA1c, is associated with an increased risk of a motor vehicle crash.

## Editors' Summary

**Background:**

Around 8% of the US population has diabetes, a group of diseases in which the body cannot control levels of glucose (sugar) in the blood. It can lead to serious complications and premature death, but suitable treatment can control the disease and lower the risk of complications.

Type 1 diabetes occurs when the body's immune system prevents the production of insulin, the hormone that controls blood glucose. It accounts for 5%–10% of diabetes cases in adults and the vast majority of cases in childhood. Patients with type 1 diabetes need to inject insulin to survive. Type 2 diabetes is associated with older age, obesity, family history of diabetes, lack of physical activity, and race/ethnicity. As obesity rates rise worldwide, it is expected that the prevalence of type 2 diabetes will increase.

**Why Was This Study Done?:**

Some complications of diabetes affect the ability to drive safely. Prolonged periods of high blood sugar levels can damage eyesight and nerves throughout the body, resulting in pain, tingling, and reduction of feeling or muscle control. Over time, some diabetics may become unaware of the early symptoms of an abnormally low blood sugar level (hypoglycemia) that can cause confusion, clumsiness, or fainting. Severe hypoglycemia can result in seizures or a coma.

It is common for driver licensing authorities to require evidence that a diabetic person's condition is well controlled before they issue a driving license. One measure of this is the percentage of hemoglobin in their blood that has joined up with glucose, known as HbA1c. This provides a measure of average blood glucose levels over the previous 8–12 weeks. A lower reading is considered an indicator of good diabetic control, but conversely, a blood glucose level that is too low can cause hypoglycemia. Normal nondiabetic HbA1c is between 3.5% and 5.5%, but 6.5% is considered good for people with diabetes.

In this study the researchers tested whether blood glucose levels, as measured by levels of HbA1c, were statistically associated with the risk of a motor vehicle crash.

**What Did the Researchers Do and Find?:**

The authors studied 795 diabetic adults who had been in contact with the driver licensing authority in Ontario, Canada between January 1, 2005 and January 1, 2007 and for whom HbA1c levels were recorded. HbA1c levels varied between 4.4% and 14.7%.

Of the drivers considered, 57 were involved in a car crash and 738 were not. The authors found that lower HbA1c levels were associated with an increased risk of a motor vehicle crash, even when they took into account other factors such as time since diagnosis, treatment, age, age when diagnosed, and, if taking insulin, age insulin started.

The authors also found that the risk of a crash quadrupled when a driver had a history of severe hypoglycemia that required outside help and that there was an increase in risk when diabetes had first been diagnosed at an older age.

**What Do These Findings Mean?:**

The authors conclude by emphasizing the difficulty in knowing whether someone with diabetes is fit to drive. They suggest that a patient's HbA1c level is neither necessary nor sufficient to determine whether a diabetic person is fit to drive and these results, which agree with some other studies, call into question the current legal framework of the US, UK, Canada, Germany, Holland, and Australia, which single out diabetic drivers for medical review.

The finding that lower HbA1c levels are associated with an increased risk of a crash is surprising, as it suggests that a driver is less safe if they control their diabetes well. However, a statistical link does not prove that one event causes another. Unknown social or medical factors might explain the results. In this case, the authors point out that a major drawback of their study is that it is not randomized and drivers have free will in choosing how tightly to control their diabetes and also how carefully they drive. The authors considered whether time spent driving might explain the results, but discounted this for several reasons. One more plausible explanation is that intensive treatment to attain a lower HbA1c level for better general health raises the risk of hypoglycemic episodes.

**Additional Information:**

Please access these Web sites via the online version of this summary at http://dx.doi.org/10.1371/journal.pmed.1000192.

- Wikipedia includes an article on diabetes (note that Wikipedia is a free online encyclopedia that anyone can edit; available in several languages)
- The American Diabetes Association publishes information on diabetes in English and Spanish
- The American Diabetes Association also publishes information on US states regulation of drivers with diabetes
- The World Health Organization of the United Nations Diabetes Programme works to prevent diabetes, minimize complications, and maximize quality of life

## Introduction

Diabetic patients account for substantial amounts of driving. At a population disease prevalence of 5% to 7% for this diagnosis, general mobility statistics would suggest that diabetic patients drive about 250 million miles during the average day in the United States [1],[2]. Such distances are extraordinary—greater than traveling from the earth to the sun and back [3]. The exact figure could be either somewhat larger if diabetes correlates with a sedentary lifestyle that favors driving or somewhat smaller if diabetes is associated with incapacitating complications that leave the patient institutionalized [4]. The substantial driving distances are likely to continue into the future given societal reliance on road travel for work, recreation, leisure, and health care [5].

On average, a population with a large amount of driving tends to have a large number of crashes. If diabetic drivers were identical to average American adults, the baseline risk of a serious crash would be about one in 20 per year [6]. This number would amount to about five diabetic drivers killed and another 50 incapacitated each day from motor vehicle crashes in the United States. Even for individuals who crash without injuries, the event can disrupt the ideals of regular exercise, a prudent diet, work productivity, and other elements of lifestyle [7]. Impairments from retinopathy, neuropathy, and hypoglycemia might make the average diabetic driver more prone to crashing than the prevailing population average [8].

Governmental policies sometimes restrict the licenses of diabetic drivers on grounds that the disease makes the individual unfit to drive [9]. Different states in the US have different regulations, yet even permissive regions require drivers who hold commercial licenses to document glycemic control [10]–[15]. The laws are based on the theory that glycemic control predicts lower driving risk either by preventing retinopathy and other complications or by indirectly distinguishing persons who are innately conscientious [16],[17]. Guidelines in Canada state, for example, “In general, a patient is considered fit to drive if it can be demonstrated that he or she is fastidious and knowledgeable about controlling his or her blood glucose levels …” [18]. In this study we tested whether glycemic control, as measured by glycosylated hemoglobin (HbA1c), was associated with the risk of a motor vehicle crash.

## Methods

### Patient Selection

We selected all drivers reported to the Ontario Ministry of Transportation Medical Advisory Board who had an underlying diagnosis of diabetes mellitus. This population-based sampling strategy included all licensed drivers in Ontario with the accrual interval spanning from January 1, 2005 to January 1, 2007, representing all years available for analysis. Candidates were identified from mandatory annual reviews submitted by drivers who held commercial licenses or mandatory reports submitted in the aftermath of a documented motor vehicle crash. We also included all other diabetic patients reviewed for any other reason such as those appealing a license suspension or those with notifiable medical conditions reported by physicians [19]. Individuals were excluded if no HbA1c was available; otherwise, all drivers were analyzed. This study was approved by the Research Ethics Board of Sunnybrook Health Sciences Center and conducted using privacy safeguards at the Institute for Clinical Evaluative Sciences.

### Crash Outcome

We classified each individual according to the manner through which they came to the attention of the licensing authority. Individuals involved in a motor vehicle crash were defined as cases. Such cases were identified by the authorities responsible for investigating a crash. All other individuals who were not involved in a motor vehicle crash were defined as controls. Such controls are not a random sample of the population because they come to attention by reports submitted by others or because of legal requirements for having a valid driver's license. Controls ought to include all diabetic drivers who developed diabetes or obtained a license during the study period, but do not because of noncompliance with legislation or other reasons.

### Glycemic Control

We obtained the medical record of each person's diabetes care from available files. These records reflect submissions from community physicians corresponding to each patient; the accuracy of these reports has never been validated although each is submitted and signed by a licensed physician [20]. We used the hemoglobin HbA1c as the primary measure of long term blood glucose control since it reflects glycemic control over 2 to 3 mo, is widely available with a liquid chromatography assay, and is the objective standard for traffic policy decisions around the world [21],[22]. In secondary analyses we also examined the patients' degree of monitoring, total years since diagnosis of diabetes, and specific complications. These secondary analyses were conducted for exploratory purposes and did not involve statistical power calculations in advance.

### Missing Data

Missing data were handled using methods blind to outcome status. The type of diabetes was not always recorded in available documents; instead, we classified individuals on the basis of whether they had started insulin treatment before or after age 20 y. The duration of diabetes was also gauged by categorizing patients who had been on insulin for 20 y or more. Data on specific complications, monitoring, and treatments were accepted as recorded under the assumption that not documented implied not present. Information on diet, exercise, weight, compliance, alcohol, lifestyle, age of first licensing, driving patterns, commercial licenses, past infractions, diabetic education, visual acuity, and at-fault analysis was not recorded and deemed not possible to impute from sources.

### Statistical Analysis

Our primary analysis compared the mean HbA1c among cases involved in a crash to controls who were not involved in a crash using an unpaired t-test with two-tailed statistics. Logistic regression was used to quantify associations using odds ratios and adjusting for baseline confounders using a step-wise forward selection procedure (models constrained to 12 events per covariate to avoid overfitting and used the c-statistic to gauge overall accuracy) [23]. Odds ratios are good approximations of relative risk for low probability events (such as the annual risk of a crash) [24]. A nonparametric test for trend was also conducted using the Cochran-Armitage method [25]. Data validation was conducted blind to outcome to correct HbA1c values outside the plausible range (4.0%–16.0%) for magnitude anomalies (e.g., 6.5% reported as 0.65 or 0.065). The sample size was estimated to provide 80% power to detect a 0.5% difference in HbA1c between the two groups of patients.

## Results

During the 2-y study interval a total of 3,900 individuals were reported to licensing authorities, of whom 795 were diabetic patients who had HbA1c values documented. Their mean age was 52 y, 84% were men, and the average patient had about a 20-y history of diabetes (Table 1). Most patients had end organ damage including retinopathy, nephropathy, and neuropathy. About 81% were treated with insulin, 27% with oral glucose-lowering medications, and 15% with neither insulin nor an oral medication. Overall, one in six lacked hypoglycemic awareness and one-third had a history of hypoglycemia that required outside assistance. The spread of HbA1c values was remarkable, ranging from 4.4% to 14.7%.

**Table 1:** Patient characteristics.

Characteristic	Feature	Crash (*n* = 57)	Control (*n* = 738)
Age	Mean years	50 (15)	52 (14)
Sex	Female	13 (23)	111 (15)
	Male	44 (77)	627 (85)
Age at diagnosis	Mean years	26 (16)	32 (16)
Age insulin started	Mean years	29 (19)	34 (18)
Extent	Insulin started<age 20 y	19 (40)	157 (26)
	Duration of insulin treatment ≥20 y	21 (43)	210 (35)
Comorbidities	Hypertension	42 (74)	453 (61)
	Retinopathy	44 (77)	604 (82)
	Nephropathy	40 (70)	590 (80)
	Neuropathy	46 (81)	632 (86)
	Stroke	4 (7)	33 (4)
	Coronary artery disease^a^	5 (9)	61 (8)
Hypoglycemia	Symptom awareness of hypoglycemia^b^	49 (86)	607 (82)
	Severe hypoglycemia in past 2 y^c^	34 (60)	200 (27)
Glucose monitoring	Computerized logs	13 (23)	90 (12)
	Handwritten logs	43 (75)	478 (65)
	Checks at least twice daily	48 (84)	576 (78)
Treatment	Insulin	47 (82)	593 (80)
	Oral hypoglycemic	21 (37)	197 (27)
	Both	20 (35)	165 (22)
	Neither	9 (15)	113 (15)
	Additional other medications (≥1)	33 (58)	481 (65)
	Three or more other medications (≥3)	13 (23)	187 (25)

Data are count (percentage) except where noted as mean (standard deviation).

a Includes myocardial infarction.

b Includes sweating or any other signal symptom.

c Defined as requiring outside assistance.

**Figure 1:**
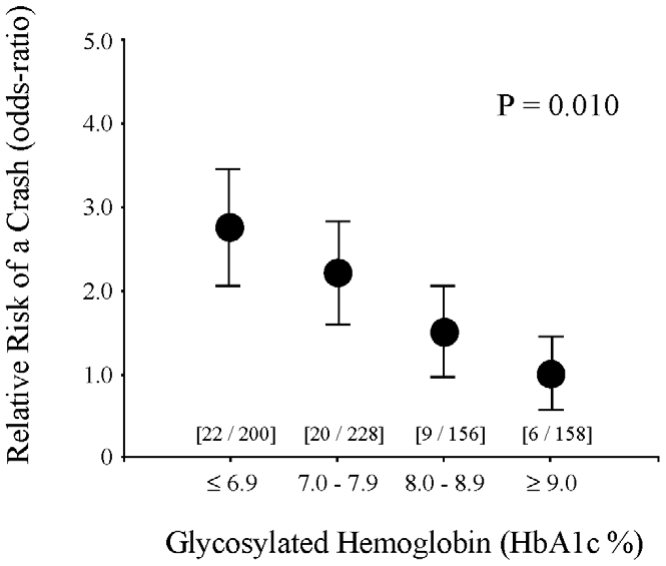
Glycemic control and risk of a motor vehicle crash. Relative risk of a motor vehicle crash for drivers at different levels of glycemic control. *x*-Axis shows glycemic control as measured by glycosylated hemoglobin concentration and grouped into approximate quartiles. Data in square brackets show individuals in each group as [number of cases/number of controls]. *y*-Axis shows relative risk of a crash expressed in odds-ratio calibrated using the top glycemic quartile as referent. Solid circles indicate point-estimates and vertical lines indicate standard error bars. *p*-Value tests for trend across all four quartiles. Overall results show a correlation between lower HbA1c levels and higher relative risk of a crash with no evidence of a U-shaped relationship.

Overall, 57 patients were involved in a crash (cases) and 738 were not involved in a crash (controls). In keeping with a potential adverse association, the mean HbA1c was lower among those who crashed than controls (7.4% versus 7.9%, *p* = 0.019). This association was equivalent to a 26% increase in the risk of a crash for each 1% reduction in HbA1c (odds ratio = 1.26, 95% confidence interval 1.03–1.54). The finding was evident across the range of HbA1c values and suggested that the risk of a crash in the bottom quartile was more than twice the risk in the top quartile (Figure 1). The absolute difference amounted to a net increase of 29 total crashes (95% confidence interval 16–46) had the risk in the highest quartile extended to all other quartiles.

**Figure 2:**
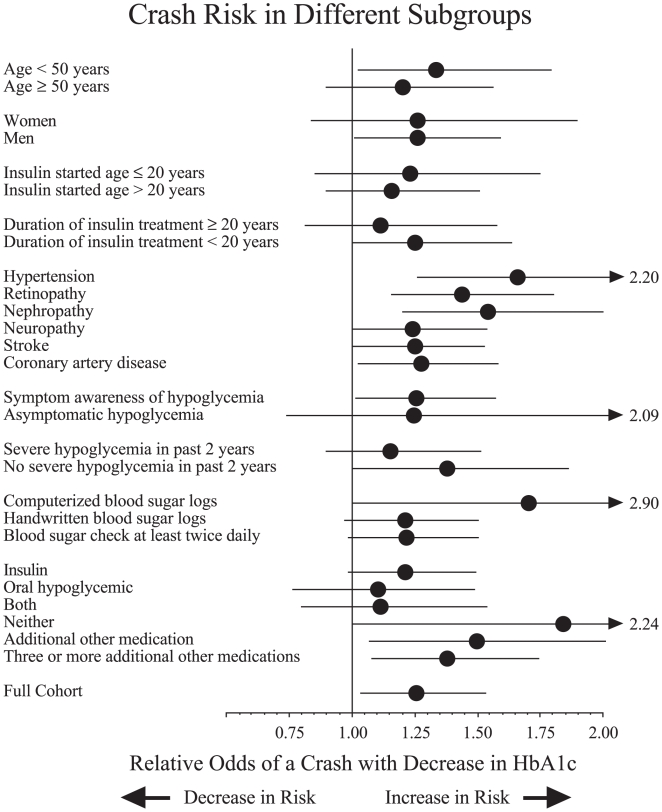
Crash risk in different subgroups. Each analysis examines correlation of lower HbA1c levels with higher risk of a crash. Results expressed as odds ratio (solid circle) and 95% confidence interval (horizontal line) per 1% point decrease in HbA1c. Analyses of chronic complication subgroups exclude patients reporting corresponding symptom. Results for full cohort appear at bottom and show an odds ratio of 1.26 with 95% confidence interval 1.03–1.54.

The observed association between low HbA1c values and increased crash risks tended to be consistent for patients with different characteristics (Figure 2). The risk was observed for patients with longer and shorter durations of diabetes, regardless of whether measured as time since diagnosis or time since starting insulin. Moreover, the risk was observed for those treated with insulin, oral hypoglycemics, both, or neither. In addition, the risk extended to those with no mention of severe hypoglycemia, hypoglycemic unawareness, or other specific chronic complications. The largest single anomaly (yet not statistically significant and overlapping the main analysis) was the subgroup not treated with insulin or oral hypoglycemic medications.

The observed association between low HbA1c values and increased crash risks persisted when adjusted for potential confounders. Analyses adjusting for age yielded approximately the same increase in the relative risk of a crash for each 1% reduction in HbA1c (odds ratio = 1.27, 95% confidence interval 1.04–1.55). Similarly, analyses adjusting for age, age at diagnosis, and age when insulin started also yielded a comparable increase in the risk of a crash (odds ratio = 1.26, 95% confidence interval 1.00–1.58). Analyses adjusting for both age, gender, and each separate complication also yielded about a 25% increase in the risk of a crash for each 1% reduction in HbA1c (odds ratio range 1.20–1.30). None of the statistical models yielded a contrary result although results in some models were not statistically significant.

Two other patient characteristics were independent risk factors for a crash. A history of severe hypoglycemia that required outside help was associated with about a 4-fold increase in risk (odds ratio = 4.07, 95% confidence interval 2.35–7.04). In addition, older age of diabetes diagnosis (expressed as increase per decade) was also associated with an increase in risk (odds ratio = 1.29, 95% confidence interval 1.07–1.57). No other baseline characteristic (Table 1) was a significant predictor of risk in univariate analyses. Multivariate analysis that included both severe hypoglycemia requiring outside help and age at diabetes diagnosis had a mid-range overall accuracy (c-statistic = 0.65) and showed a persistent association of HbA1c with crash risk (odds ratio = 1.25, 95% confidence interval 1.02–1.55).

## Discussion

We studied a selected sample of diabetic adults driving during a 2-y interval using a population-based approach. The main finding was that lower HbA1c levels were associated with an increased risk of a motor vehicle crash. The adverse association was observed across the range of HbA1c values, persisted after adjustment for independent confounders, yet was not as large as the relative risk associated with a history of severe hypoglycemia requiring outside assistance. The attributable risk was substantial, so that eliminating the association by extrapolating the risk observed at the highest HbA1c quartile to all drivers at all HbA1c quartiles would have eliminated about half of all observed crashes. These findings are difficult to explain with random chance, reverse-causality, or simple reporting bias.

A major limitation of our research relates to the nonrandomized design and sample selection. That is, adults with diabetes self-select how to control their glucose as well as how to drive a vehicle. One explanation for the association, therefore, could be that those who are stringent about controlling their blood glucose are paradoxically more careless about driving a vehicle. Another explanation could be that tightly controlled patients drive in more dangerous settings. A third explanation could be that unreported alcohol consumption influences both driving risk and glucose control (e.g., impaired liver glucogenesis). Many other biases are possible including Berkson's paradox, Neyman Bias, Hawthorn effects, restricted generalizability, imperfect compliance with the law, and spectrum bias [19],[26]. These limitations are unavoidable in trauma research except for studies that focus on volunteer samples, unnatural tasks, or hypothetical risks [27].

We have no data on baseline time spent driving, yet such data are unlikely to explain our findings. First, all individuals maintained valid licenses, remained active in the community, and were at risk for a crash. Second, no prior study shows diabetic adults drive substantially more than the prevailing average (or that small differences in HbA1c predict large differences in driving time) [28]. Third, research in other domains indicates time spent driving is a poor predictor of crash risk; for example, teenagers account for a large number of crashes despite a small amount of time spent driving and senior citizens have a heightened risk primarily explained by the very low distance drivers [29],[30]. No surprise, therefore, that license regulations account for fitness to drive but have no restrictions based on the amount of driving the person intends.

Our findings join a growing and contentious literature correlating low HbA1c values with adverse consequences in adults with diabetes mellitus. For example, three recent randomized trials found that intensive treatment regimens led to both lower HbA1c values and an increased incidence of severe hypoglycemia among diabetic patients [31]–[33]. These trials and our study do not prove that striving for a normal HbA1c is harmful; instead, the adverse association might indicate that customary treatments for achieving euglycemia are inexact and potentially hazardous to high level cognitive behavior [34]–[37]. Many patients, furthermore, are aware of their HbA1c results so that a double-blinded trial becomes unfeasible and susceptible to subtle confounders. Such behavioral factors are germane in clinical research since patients with a normalized HbA1c might develop a false sense of security whereas those with a high HbA1c might abandon their activities and ironically become protected from mobility related injury [38].

The basic implication of our study is to underscore the difficulty in judging fitness-to-drive in adults with severe diabetes mellitus [39]. This pitfall calls into question traffic laws that prevail in the United States, United Kingdom, Canada, Germany, Holland, Australia, and other countries that single out diabetic patients for specialized review. At a minimum, the data suggest that a patient's HbA1c level is neither necessary nor sufficient for determining fitness-to-drive. Whether a comprehensive medical review, functional performance assessment, formal driving test, detailed record of hypoglycemia episodes, or other measure could be more accurate and cost-effective remains a topic for future research. Unfortunately, most other measures of diabetes control are based on self-report that can be easily denied when applying for a driving license.

## Abbreviations

HbA1c: glycosylated hemoglobin
